# From Self to Nonself: The Nonself Theory

**DOI:** 10.3389/fpsyg.2016.00124

**Published:** 2016-02-04

**Authors:** Yung-Jong Shiah

**Affiliations:** Graduate Institute of Counseling Psychology and Rehabilitation Counseling, National Kaohsiung Normal UniversityKaohsiung, Taiwan

**Keywords:** nonself, the nonself theory, Buddhism, compassion, meditation

## Abstract

The maintenance/strength of self is a very core concept in Western psychology and is particularly relevant to egoism, a process that draws on the hedonic principle in pursuit of desires. Contrary to this and based on Buddhism, a nonself-cultivating process aims to minimize or extinguish the self and avoid desires, leading to egolessness or selflessness. The purpose of this paper is to present the Nonself Theory (NT). The universal Mandala Model of Self (MMS) was developed to describe the well-functioning self in various cultures. The end goal of the self is to attain authentic and durable happiness. Given that the nonself is considered a well-functioning self, the MMS is suitable for constructing the NT. The ego and nonself aspects of psychological self-functioning and their underlying processes are compared, drawing on the four concepts of the MMS: biology, ideal person, knowledge/wisdom and action. The ego engages in psychological activities to strengthen the self, applying the hedonic principle of seeking desire-driven pleasure. In contrast, a nonself approach involves execution of the self-cultivation principle, which involves three ways: giving up desires, displaying compassion, practicing meditation and seeking understanding Buddhist wisdom. These three ways have the goal of seeing through and overcoming the illusion of the self to achieve a deep transformation integrally connected to the experience of eliminating the sense of self and its psychological structures. In addition, the NT provides a comprehensive framework to account for nonself-plus-compassion-related activities or experiences such as altruism, mindfulness, mediation, mysterious/peak experiences, elimination of death anxiety and moral conduct. The NT offers possible answers that might lead to a more comprehensive understanding of human beings and the deeper meaning of life, toward the ultimate goal of a well-functioning self. An examination of possible clinical applications and theoretical directions for future research in nonself psychology are provided.

## Two types of self

Traditionally, Western psychology has attempted to understand the psychological functioning of the self from an individualistic perspective (Triandis and Gelfand, 1998; Triandis, 2001), emphasizing the need to satisfy, maintain and strengthen the self (Greenberg et al., 1990; Burke et al., 2010). There are numerous formulations of the self in Western psychology, and many of these are constructed on the basis of their being a definite “I” entity (Shonin et al., 2014). Thus, psychology has found an important role for the “self” in an abundance of subject-hyphen- predicate relations (Klein, 2014) (e.g., self-affirmation, self-awareness, self-comparison, self-concept, self-consistent, self-control, self-efficacy, self-esteem, self-determination, self-fulfillment, self-handicapping, self-image, self-identity, self-perception, self-regulation, self-reference). The origin of the concept of the individualistic view of self can be traced to early Christianity. Protestantism is considered to be the denomination most strongly related to American culture and, more specifically, to the American individualistic view of self (Oyserman et al., 2002; Cohen and Hill, 2007).

In the East, for more than 2500 years Buddhists have adopted a distinctive approach to the concept of the self (Kelly, 2008). Buddhism is commonly defined as including Southeast Asian Theravada Buddhism, East Asian Mahayana Buddhism, Indo-Tibetan Mahayana, and Vajrayana Buddhism (Wallace and Shapiro, 2006; Shonin et al., 2014). The ultimate aim of these schools is to overcome the pain and emotional disturbances caused by life's difficulties, challenges, and stressors (Shiah and Yit, 2012). The Buddha's teachings are aimed at attaining an authentic, durable happiness by cultivating a transition from the self state to the nonself state (Dalai Lama, 1995a, 2005). Buddhism holds that personal identity is delusional (Giles, 1993), that each of us is a self that turns out to not actually exist (Dalai Lama, 1995b, 2005). Clinging to or being obsessed with the delusional self is the major cause of suffering (Dalai Lama, 1995a). In contrast to the concept of the self, the eternal goal of Buddhists is *nirvana* (Dalai Lama, 2005), a state of nonself that involves a process of renouncing worldly things, particularly those for which attractiveness springs from egoism and desires, while maintaining or elevating the self, or *atama-graha* (Hwang and Chang, 2009; MacKenzie, 2010). This process leads to *nirvana or* the state of nonself, a state of total liberation (Tsong-Kha-Pa, 2000; Shonin et al., 2014). However, the total liberation state concept in Buddhism is complex (Tsong-Kha-Pa, 2000) and transcends psychology. Nonetheless, the nonself state has consequences in the psychological domain, such as authentic and durable happiness (Dambrun and Ricard, 2011), and only these psychological consequences are discussed in the present paper. The generally agreed upon estimate of the number of Buddhists is around 350 million (6% of the world's population), most living in the East (Number of Buddhists worldwide, 2015). In the West, one in four British adults practices meditation, and over 20 million people practice it in America (6.5% of the population) (Shonin et al., 2014). Over the past 30 years, a growing number of psychotherapists, counselors and mental health workers have been engaged in various forms of Buddhist psychotherapy (Michalon, 2001; Kelly, 2008; Shonin et al., 2014; Murguia and Diaz, 2015), such as compassion-based therapy (Gilbert, 2009; Galante et al., 2014; Shonin et al., 2015), Buddhism-based grief therapy (Wada and Park, 2009; Lee et al., 2015a) and mindfulness-based techniques (Khoury et al., 2013, 2015). It is of academic interest to hypothesize that Buddhism provides an alternative perspective on the self and ways to manage one's daily life. In fact, there have been many studies attempting to link Buddhism to psychology (Wallace and Shapiro, 2006) and psychotherapy (Shonin et al., 2014), the majority of which have focused on meditation and its effects, such as increased emotional stability (Lee et al., 2015b), heightened positive emotion (Fredrickson et al., 2008), mindfulness (Brown and Ryan, 2003; Khoury et al., 2015) and improved attention (Sedlmeier et al., 2012; Lippelt et al., 2014). There have been few studies on theories directly relating Buddhist core teachings to nonself. Given that the most central concept in Buddhism is the nonself (Dalai Lama, 1995a; Hwang and Chang, 2009; Albahari, 2014), the purpose of this paper is to present a theory, named the Nonself Theory (NT), based on Buddhist teachings. The NT integrates different disciplines ranging from Western psychology to Buddhism.

The universal Mandala Model of Self (MMS) was developed to describe the well-functioning self in various cultures (Hwang, 2011). Given that the nonself is considered a well-functioning self, the MMS is a suitable basis for constructing the NT. In this paper, I compare the self and nonself aspects of psychological self-functioning, drawing on the four concepts of the MMS: biology, ideal person, knowledge/wisdom and action. An examination of possible applications and theoretical directions for future research are provided at the end.

### Definitions of the self and the nonself

The self is the locus of empirical experience and it can take various actions depending on the social context (Hwang, 2011). The present theoretical model proposes two kinds of self, namely the self and the nonself. Two principal dimensions underline these two kinds of self: egoism and no self. I propose that these two kinds of self are end points on a continuum. Each of us falls at a certain place on this continuum. I define egoism as a desire-driven sense of self (Albahari, 2014). As long as we believe a self belongs to us, we are each an example of egoism (Dalai Lama, 1995a). The psychological functioning of egoism is characterized by such attributes as biased self-interest, self-centeredness and egocentrism (Dambrun and Ricard, 2011). Thus, it is assumed that egoism is a central point of reference for psychological activities, following the hedonic principle of pursuing stimulus-driven pleasure. The strong importance given to egoism emerges mainly from its connection with self-centeredness. Egoism is inclined to increased extent to which the individual considers that his or her own condition is more important than that of others and takes unquestioned priority.

On the contrary, as noted before, a state of nonself involves renunciation of worldly things, particularly those that are attractive because of egoism and desirse. Personal identity or the self is delusional (Giles, 1993; Joshanloo, 2014); such a self is assumed to not actually exist or not to be permanent (Dalai Lama, 1995b). The Dalai Lama (2005) asserted that the term *nonself* refers to the realization that the self or the I lacks intrinsic existence.

## Mandala model of self and theory of nonself

### Mandala model of self (MMS)

**Figure 1:**
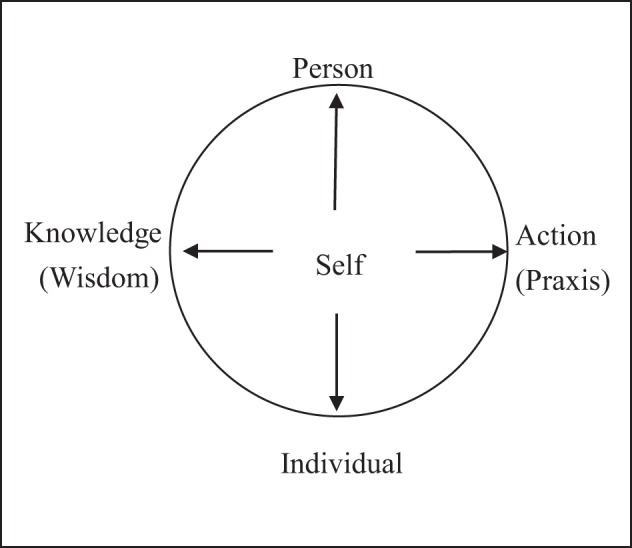
**Mandala model of self**.

The MMS was inspired by Buddhism and constructed to provide a universal model that describes the well-functioning self in all cultures (Hwang, 2011). The end goal of the self in all cultures is to authentic and durable happiness (Dambrun and Ricard, 2011; Johnson, 2015). In this model, an individual living in his or her life-world is represented by a circle inside a square (see Figure 1). Jaffe (1964) noted that alchemists played an important role in Europe around 1000 A.D, when various sects appeared. European alchemist tradition or practice is to purify, perfect and complete certain objects. These sects sought to integrate mind and body, creating many names and symbols to denote this integration. One of these symbols was the quadrature circle.

Jaffe (1964) indicated that the circle represents the most important aspect of life, which she calls a well-functioning self. It exists in a wide range of contexts, such as sun worship by primitive peoples, modern religions, myths and dreams, the mandala of the Tibetan lamas, and the planar graphs of secular and sacred architecture in every civilization. The square, in contrast, symbolizes secularism, the flesh, and objective reality. Therefore, the mandala can be viewed as the symbol for a prototype or deep structure of the self.

In Figure 1, the self resides inside a circle located between two bi-directional arrows: One end of the horizontal arrow points to “action” or “praxis”; the other end points to “wisdom” or “knowledge.” The top of the vertical arrow points to “person,” and the bottom points to “individual.” All four of these terms are located outside the circle but within the square. This arrangement of the concepts means that the self is being influenced by forces from the individual's external environment.

Anthropologist Grace Harris (1989) proposed that the terms “person,” “self,” and “individual” have very different meanings in the Western academic tradition. This tradition treats “individual” as a biological term; individual human beings are considered the same as other creatures. “Person” is a sociological or cultural term; the individual is conceptualized as an agent-in-society who adopts a certain role in the social order and plans a series of actions to achieve a particular goal. Every culture has its own definitions of appropriate and permitted behavior. Each class of actions is endowed with a specific meaning and value that is transmitted to the individual through various channels of socialization.

“Self” is a psychological term. In the conceptual framework of Figure 1, as noted earlier, the self is the locus of empirical experience, and it takes various actions depending on the social context. It also engages in reflexivity when blocked from attaining its goals. According to Giddens' (1984, 1993) Structuration Theory, the self as the subject of agency is endowed with two important capabilities: reflexivity and knowledgability. Reflexivity is a recursive loop in which a thing becomes self-referential (Sundararajan, 2008). Self-reflexivity is a process and a kind of action in terms of the ability to monitor and explain its actions. Knowledgeability is the ability of the self to memorize, store, and organize various forms of knowledge into a well-integrated system that guides reflexivity and action. An individual's self-identity and social-identity have very important implications for reflexivity. When individuals intend to act, their decisions may be influenced by all four forces in Figure 1, especially if they identify with a particular social role. On the one hand, individuals must think about how to act as socialized persons. On the other hand, as biological entities they are pushed by various desires. When they take action and encounter barriers, they may engage in action-oriented reflexivity (Eckensberger, 1990, 1996, 2012), using the information available in their stock of knowledge. If the barrier persists, they may take further steps to search for a solution from their social stock of knowledge. If they identify with a particular social group, they may communicate with other group members, thereby constructing a mutually shared social reality that may be plagued by specific problems. Individuals may then have to search their stock of knowledge to find solutions to these problems on behalf of the group.

The wisdom contained in the personal stock of knowledge can lead individuals to act intelligently in various social contexts. According to the theoretical model shown in Figure 1, the social praxis of the self in a given context is pulled by two forces—the person as a social agent and the individual as an organism. To act in a manner accepted by society, individuals who want to satisfy their own desires must learn how to act in accordance with the sociomoral order, using the process of socialization guided by their wisdom. According to the MMS (Hwang, 2011), one important characteristic of individuals is the ability to engage in agency-oriented reflexivity on the meaning of life. People in different cultures exhibit different kinds of wisdom when they think about spirituality, the meaning of their life, and their morality. Individuals' attempts to define their own moral conscience create a normative wisdom that circulates within the society. This wisdom can be used as material for thinking or for meta-ethical reflexivity. Because the MMS proposes a dynamic interaction involving self, individual, knowledge/wisdom and action/praxis, I draw on these four concepts to describe the process of self-cultivation that moves one from self to nonself. This description is based on Buddhist teachings as well as Western psychology.

### From self to nonself

**Figure 2:**
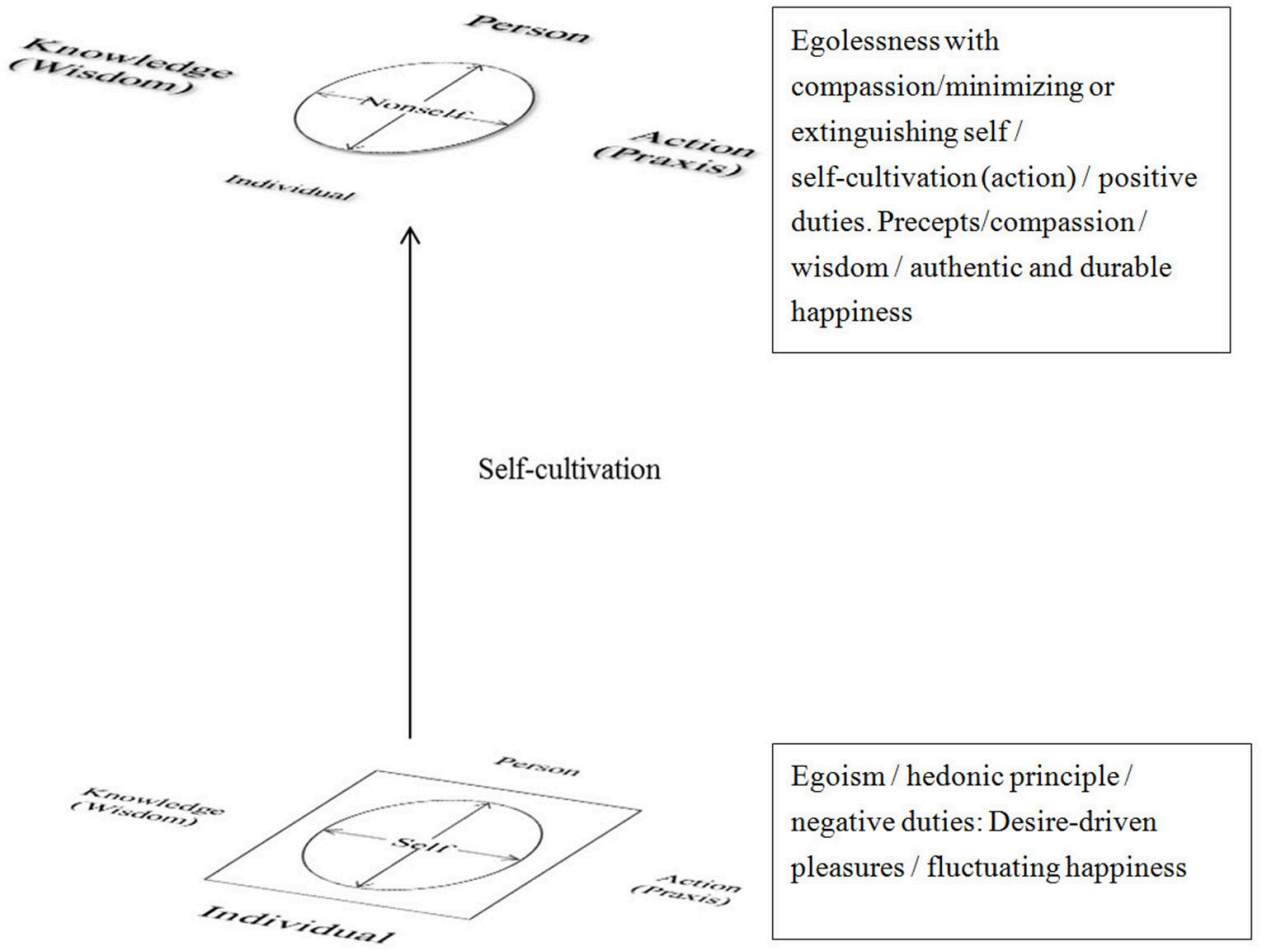
**Nonself thoery**.

In the NT (see Figure 2), there is a single continuum from the bottom (self) to the top (nonself). In this section, we ask what Buddhism has to say about how and why our brains change, and how and why complex and pervasive patterns of desire-driven emotion and action are eliminated, resulting in a perceived identity or the self. How Buddhism helps us to achieve the nonself state is also addressed. Buddhism suggests that we apply the self-cultivation principle by obeying certain precepts, practicing compassion, and absorbing wisdom. The aim of all this is to see through and overcome the delusion of the self. The nonself state is authentic and durable happiness.

#### The cause of the delusion of self leading to suffering: the biological individual self

According to the NT (see Figure 2), only the top of nonself circle is not a delusion. As noted before, a state of nonself involves renunciation of worldly things, particularly those that are attractive because of egoism and desires. Thus, there is no square on the top of nonself. In general, one perceives the self via first-person subjectivity: the person has the experience rather than the object (Klein, 2014). The self is formed from perceptions, desires, needs and psychological functions of the biological individual; these functions are conation, motivation, attention, cognition, emotion and behavior (Dalai Lama, 1995a; Dambrun and Ricard, 2011; Shiah and Yit, 2012). The biological individual initially gives the self a personal identity or uniqueness, a feeling of ownership of various phenomena in the mind, body and external world (Albahari, 2006). This sense of self is part of a hedonic principle and a deep-seated, reflexive false belief (Albahari, 2014). The hedonic principle codifies a very intense desire-driven way of relating to objects, events, situations, substances, the body, and even life itself (Leifer, 1999). The self is powerful in shaping our lives and self-identity. In this paper, self-identity also refers to egoism.

#### Why does egoism cause suffering?

Western psychologists recognize egoism as one of the basic motivations of the human mind: It is desire for pleasure and aversion to pain (Leifer, 1999). In Buddhism, as long as we have the self, we will be egoistic (Dalai Lama, 1995a). Why do egoism and application of the hedonic principle cause pain? There are four explanations. In Buddhism, suffering is caused by desire (Leifer, 1999; Dalai Lama, 2001). Clinging to the self is mainly an attempt to fulfill desires (Dalai Lama, 1995a). When we crave something pleasant, we tend to reject its opposite. The self-structure is completely devoted to mental “defilements,” such as preferences and aversions. They make us attached to things being one way rather than another, causing us to suffer if the desire is frustrated (Albahari, 2014). The second explanation is that these desire-driven pleasures are contingent upon the appearance or disappearance of specific stimuli that can potentially satisfy egoistic desires. In general, such pleasures are unreliably contingent on stimuli from the environment, from interactions with other people, or from various kinds of physical and mental activity (Dalai Lama, 1995a). When the stimulus is present, the pleasure associated with it appears. However, as soon as the stimulus disappears or is supplanted by a new stimulus, the positive feeling fades, which causes hedonic adaptation, the process by which people quickly become accustomed to the positive (or negative) effects of new stimuli and eventually return to their baseline level of happiness (Dambrun and Ricard, 2011). Therefore, the pleasure generated by applying the hedonic principle is short-lived and unstable (Steger et al., 2008; Hallam et al., 2014; Huta and Waterman, 2014). Regardless of whether the feeling is negative or positive, what is important is that the feeling can be seen as an object to be observed but that does not belong to us (Dalai Lama, 1995a). Thirdly, in Buddhism (as we discuss more fully later) egoism is not real. Thus, pursuing the delusion of the self ends up being in vain. This last proposition is grounded in Western psychology, which focuses on understanding the psychological functions associated with how the self deals with threats to the life of the individual. Undoubtedly, death is the greatest inevitable threat and a challenge to the self or the identity of a human being. The fact that we all will die means that the self will disappear and shows that life is fragile. The self needs to find a way to cope with death.

Arguably the most sophisticated theory on how the self copes with death is Terror Management Theory (TMT) (Greenberg et al., 1986, 1992), which defines self-esteem as a feeling of *significance*, a sense of significant meaning employed to defend against the fear of death. TMT treats self-esteem as a cultural construct with a variety of perceived meanings. These meanings importantly provide information about the presence of reliable patterns and coherence in the environment (Heintzelman and King, 2014), helping one cope with life's adversities (Park, 2010). TMT suggests that self-esteem is a sense of personal worth that is derived from the belief in the validity of the worldview of one's culture and from living up to the standards that are part of that worldview (Pyszczynski et al., 2004). Feeling significant means seeing oneself as a valuable contributor to a meaningful universe and that one's life has both meaning and value. According to TMT, avoidance of death anxiety is the strongest human motive and it motivates all human activities. It triggers defenses against death anxiety that are erected by maintaining one's worldview and self-esteem (Burke et al., 2010). In fact, the threat of death never disappears and comes up in a variety forms all the time. Therefore, as soon as self-esteem is attained, a new threat may supplant the initial one. This replacement of the initial stimulus by another one might cancel the positive effect of the self-esteem generated by the first stimulus. In other words, we need endless action to maintain and strengthen the self so it can cope with death anxiety or unhappiness. We might find happiness sometimes, but we need to endlessly reboot it. Death anxiety is accompanied by endless negative emotion, which can take the form of, for example, anger, greed, jealousy, anxiety, depression, hatred, pride or fear of unhappiness.

#### Wisdom/knowledge, person and action conquering the individual: three ways to minimize the sense of self

The Buddha's teachings are aimed at helping us escape from the delusion of the self and attain a nonself state (Dalai Lama, 1995a, 2005). The way to achieve this goal is to implement a three-way process (Dalai Lama, 1995a; Kelly, 2008; Albahari, 2014; Shonin et al., 2014) that specifically is intended to reveal to one the emptiness of desires and of the self (Alt, 1980). In the present paper, these three ways are considered as the components of a self-cultivation process. Specifically, we describe the interaction of four components of the self as defined in the MMS in the context of Buddhism. The process uproots the deep-seated and reflexive false belief that one is a self, and it re-aligns and integrates one's emotional, cognitive and behavioral dispositions in accordance with the correct belief that there is no such self (Albahari, 2014). Note that eliminating the existence of the self does not entail denying the reality of every feature ascribed to the self, a consequence that some would find implausible. Non-delusional feature ascribed to the self can survive dissolution of the self-delusion, hence the phrase “eliminating the existence of self” should be read as “losing the sense of those delusional features ascribed to the self” (Albahari, 2014).

According to the MMS, the first and second ways of the process are actions aimed at the cessation of desire, and the second way (meditation) draws on the concepts of person and action. The third way draws on the concept of wisdom/knowledge. These three ways aim to annihilate the individual as defined in the MMS, especially its biological and psychological desires.

The first way is to renounce the worldly things that we desire (Dalai Lama, 2001). The psychological function of egoism affects the biological individual via application of the hedonic principle, which states that individuals are motivated to obtain pleasure and avoid displeasure. Egoistic behavior frequently is a response to impulses toward pleasurable stimuli and away from unpleasant ones. As a result, there is a strong focus on pleasurable stimuli, leading to satisfaction. This process mainly creates a sense of identity, which is a delusion (Dalai Lama, 1995a).

The link between egoism and desire is very robust and reflexive (Albahari, 2014). Obeying precepts is a self-cultivation process used to break this robust and reflexive link between the self and desire directly. Precepts define ways to give up desire-drive conations and actions that involve the nonself performing negative and positive duties. Negative duties are egoistic behaviors that follow from obeying the principle of refraining from harming and injuring others and the associated laws. Positive duties, which involve conation, motivation, attention, cognition, and emotion, as well as behavior, obey the precepts of meditation and wisdom.

In the first stage of the path to the nonself, three main strategies are used to turn off desire. The most basic and easiest strategy to execute is to follow the precept rules that specify the behaviors that are not allowed. Given that desires are innately linked to the biological individual, the desire conation inevitably and reflexively comes out when we intentionally cease desire-driven behavior. The desire conation is like a seed from which motivation, attention, emotion and behavior spring (Dalai Lama, 1995a; Sheng Yen, 1999). It is strongly stressed in Buddhism that the steps designed to stop or extinguish the desire conation must be executed fully. The other two methods to cause the cessation of the desire-driven connation (meditation and wisdom/knowledge) are discussed later. It should be stressed that completely eliminating a desire-driven connation is a very challenging and extremely hard first step on the path to the nonself.

Precept is an umbrella term that subsumes five major precepts (Sheng Yen, 1999; Salgado, 2004; Ariyabuddhiphongs and Jaiwong, 2010). The first precept is to refrain from killing. The central tenet of Buddhism is non-harming, as Buddhism teaches the sanctity and equality of all life—humans, animals, plants, and even the non-biological environment (which is assumed to be living). The second precept is to refrain from taking things not given to you. Aside from outright stealing, this also includes consuming more than necessary and wasting resources. The third precept is to refrain from sexual misconduct. This includes sexual assaults, infidelity, promiscuity, and for Buddhists, noncelibacy. The fourth precept is to refrain from speaking falsehoods. The fifth precept is to refrain from substance abuse. Buddhism emphasizes wisdom and clarity of mind. It teaches us to “look within” to find our Buddha nature. Substances such as alcohol, cigarettes, and drugs cloud our minds and thus impair our ability to practice precepts. Furthermore, when using such substances, we are prone to disobey the previous four precepts.

The second method to eliminate desire-driven conation and behavior is meditation. It has two additional purposes. The second, which applies specifically to compassion meditation and death mediation, is to facilitate the dissolving of the self-process via loving kindness. The third, which is described in a later section, is to acquire clear insight into the reality of all things. In Buddhism, obeying the precepts and meditating are the best ways to get a clear insight into this reality (Dalai Lama, 1995a). Meditation is not restricted to specific locations and times.

In general, two kinds of meditation are especially useful in achieving the first and third goals. They are focused attention (FA) and open monitoring (OM) meditation (Lutz et al., 2008). FA meditation entails voluntarily focusing attention on a chosen object in a sustained fashion. OM meditation involves non-reactively monitoring the content of one's experience from moment to moment, primarily to recognize the nature of emotional and cognitive patterns. There are three main purposes of FA and OA meditation. The first purpose is to cultivate our conation, motivation, attention, and emotion so that we can renounce the worldly things that we desire. The second purpose is to induce a quiet consciousness (Cahn and Polich, 2006; Sedlmeier et al., 2012) as indicated by low-frequency (alpha) electroencephalography (EEG) rhythms, thereby sharpening perception of the sensations, conations, motivation, attention, and emotion. It is natural for one to involuntarily or voluntarily link one's conation/behavior to one's desires. Practicing FA and OA can gradually minimize and eventually break these links between conation flow/behavior and desires (Dalai Lama, 1995a). This practice gradually increases insight into the reality of all things and causes a loss of the sense of self. The third purpose is to gradually minimize the conation flow and behaviors that are linked to desire. One also gets clear insight into the unreality of the body and all things (Dalai Lama, 1995a).

Compassion is habitually having the conation that all living beings are inextricably interconnected (Hofmann et al., 2011). Specially, compassion meditation involves practicing loving-kindness and compassion; sympathetic joy—joy in another's joy, the opposite of schadenfreude; and equanimity—being calm and even-tempered (Buddhaghosa, 1975; Dalai Lama, 1995b). These four components are called *brahma viharas*, sublime states, also known as noble and divine abodes, or “immeasurables,” that can be cultivated. They are described in the Visuddhimagga, an influential Buddhist text (Buddhaghosa, 1975). This practice does not require concentration on particular objects, memories or images, although in other meditations that are also part of their long-term training, practitioners focus on particular persons or groups of beings. Compassion meditation induces an arousal of consciousness that is indicated by high-frequency (gamma) EEG rhythms in long-term Buddhist meditators (Lutz et al., 2004). In fact, according to this definition of compassion, obeying the precepts is actually practicing compassion (Sheng Yen, 1999). This is because in obeying the precepts one does not do bad things and is selfless. Not doing self-centered things is doing good things and is compassionate. Thus, obeying precepts is considered a kind of compassion meditation (Sheng Yen, 1999; Tsong-Kha-Pa, 2000). Most importantly, compassion meditation stops the conation and behavior that are linked to desire.

Death meditation has two forms: Asubha meditation and death contemplation (Shiah and Yit, 2012). In Asubha meditation, one visualizes the decomposition of a dead body. Its goal is to give one insight into the true nature of the body, the self and desire, i.e., that they are unpleasant, disgusting, ugly, impermanent and cause suffering. Another goal is to create insight into the impermanence and emptiness of body and self. As will be discussed in a later section, this meditation is considered conducive to overcoming desire and lust (Shiah and Yit, 2012). Death contemplation is to frequently think intensively that death will someday come upon one. These two death meditations aim to reduce desire and attachment to the body. It is the contemplation of death that helps destroy our infatuation with and desire for pleasure.

The third way is to obtain the Buddha's wisdom/knowledge through practicing the meta-ethical reflexivity that guides the actions of an ideal person. The major goal of the Buddha's teachings is for one to learn the reality of emptiness and to increase compassion. Ignorance of Buddhist wisdom leads to enhancement of the self, which in turn leads to suffering (Dalai Lama, 1995a). This suffering and pain become so great that the person tries to get rid of it (Alt, 1980).

There are four major components of the Buddha's wisdom/knowledge (Leifer, 1999; Kelly, 2008; Nickerson and Hinton, 2011; Albahari, 2014): impermanence, suffering, the doctrine of dependent origination, and emptiness. Note that these ultimate realities can be observed in all things, especially during meditation (Dalai Lama, 1995a).

The truth of impermanence means that all compound objects, including ourselves, eventually decay, disintegrate, and die (Rinpoche, 1998). Thus, the reality of the body and all things is emptiness leading to impermanence (Rinpoche, 1998; Dalai Lama, 2005). The universal law of impermanence applies as much to psychological phenomena, such as thoughts feelings, and perceptions, as it does to material phenomena, both animate (e.g., the birth, living and death of sentient beings) and inanimate (Dalai Lama, 1995a; Shonin et al., 2014). The concept of impermanence is related to suffering, because suffering arises from craving things we desire; this craving originates in the self (Dalai Lama, 1995a). All desires give rise to much suffering when the self is attached to them. This is because they are changeable and impermanent, i.e., a reflection of emptiness. Again, obeying precepts and meditating keep the self from craving and stop suffering. They help us understand that impermanence and the actions that arise from emptiness are all related to the nonself. Life is full of suffering. We inevitably suffer at birth and suffer from not getting what we want, suffer from getting what we don't want, suffer from getting old, suffer from getting sick, and suffer from dying. Also, the threat of one's own death causes a great deal of terror and pain, which in turn creates a strong motivation to seek meaning in death (Burke et al., 2010; Shiah and Yit, 2012). Buddhism considers death the greatest threat and suffering to human beings (Rinpoche, 1998; Shiah and Yit, 2012). In Buddhism, death serves as both a conscious and an unconscious reminder that life is finite and impermanent; recognition of this directly leads to anxiety followed by a search for the meaning of life (Shiah and Yit, 2012). In its initial stages, Buddhist theory draws heavily on the idea that this innate mechanism motivates the person to seek and be aware of the truth mentioned above about what causes our anxiety. This mechanism also motivates the person to seek a solution for reducing the anxiety, and especially for the nonself to pursue meaning. In its later development, cultivation of the transition from the self state to the nonself state was added as a way to totally conquer or eliminate death anxiety. Cultivating an awareness of the reality of death and of impermanence can resolve the suffering (Rinpoche, 1998).

The doctrines of dependent origination (causality) and karma say that each and every occurrence becomes a cause of all subsequent occurrences throughout space and time (Shonin et al., 2014; Allen et al., 2015). Everything is composite; there may be a cluster of causes for any one effect and any one cause can lead to multiple effects (Kelly, 2008). Thus, nothing has an intrinsic identity or substance. Phenomena are outside and independent of the self, and all things are in a continual process of arising and passing away (Dalai Lama, 1995a). The failure to understand these facts is the root cause of suffering (Shiah and Yit, 2012). This is because we pursue those things that our body desires. Our desires are nearly always aimed at the happiness of the self (hedonic principle), which can make a person more selfish and thereby have a negative effect on the well-being of others (Dambrun and Ricard, 2011). Buddhists argue that the desire-driven pursuit of happiness can lead to such negative emotions as cruelty, violence, pride, and greed, which in turn cause happiness to fluctuate (Dambrun and Ricard, 2011). Note that the Buddha's wisdom/knowledge can be observed and learned from obeying precepts and meditating (Dalai Lama, 1995a). Pursuing desires not only creates a lack of awareness, but it also projects onto both the self and the external world something that is not there—namely, emptiness (Dalai Lama, 1995a).

The purpose of meditation is to get rid of desires. At the same time, one is obeying precepts and practicing compassion, which makes one aware of emptiness. The essential wisdom/knowledge in Buddhist teaching consists of the reality of emptiness plus the value of compassion. Thus, compassion can be considered an authentic from of wisdom and a way to attain the state of nonself. Buddhists believe that the nonself state projects an unconditional, limitless loving kindness and compassion. Buddhists have long believed that this state conquers death anxiety (Shiah and Yit, 2012) and achieves an authentic and durable happiness (Joshanloo, 2014). The function of compassion is to minimize or extinguish the self, leading to creation of the nonself or selflessness (Shiah and Yit, 2012). Thus, the state of nonself-plus-compassion is thought to provide one with the fundamental meaning of being alive (Shiah and Yit, 2012). This is the major reason why the core teaching of Buddhism is compassion (Dalai Lama, 1995b; Wallace, 2001; Fink, 2012; Shiah and Yit, 2012; Albahari, 2014).

Because we realize the impermanence experientially, we arrive at the understanding of nonself. This is not a loss of the self. It continues to exist, but we don't see it the same way. What we gain is the true insight that the self is not the body and the mind (Dalai Lama, 1995a). Specifically, according to the MMS, reflexivity plays an important role in extinguishing the link between desire and the self. It monitors and understands its actions through a recursive loop, in which a thing becomes obeying precepts, meditating, and absorbing Buddhist wisdom. We can imagine many conations arising to link our desires to the creation of a sense of self. Reflexivity monitors these conations and breaks them down. Each breaking, which lasts only for a short “breaking-moment,” has three steps: emergence, presence, and dissolution (Nyanaponika, 1998). The conations that reflexivity monitors are linked to desires associated with the eyes, ears, nose, tongue, and the whole body. Reflexivity draws on meditation and wisdom to neutralize the five senses by keeping one aware of the desire conation without actually perceiving it (Brown and Ryan, 2003). In the meantime, our practices aimed at absorbing wisdom eventually reach a stage at which we clearly understand that everything, including the self's activities and all physical phenomena, arise, change and eventually pass away (Dalai Lama, 1995a). Finally, step by step, we attain the deep reflexivity that sees through and overcomes the delusion of the self and then dissolves the self. Thus, reflexivity is essential for maintaining meditative awareness, obeying the precepts, absorbing Buddhist wisdom, and cultivating a state of the self that is conducive to the creation of the state of nonself.

On the contrary, egoism is inclined to avoid obeying precepts, meditating and understanding all objects of ultimate reality. It tends to perform only negative duties and seeks enjoyment from sensual pleasure, such as attractive visual images, sounds, aromas, tastes, and tactile sensations. As a result, there is a strong focus on the satisfaction derived from pleasurable sensations, self-defense and egocentric biases (Dambrun and Ricard, 2011). The acquisition of material goods, financial security, power and fame may also lead to happiness, but they are too transient. Desire is nearly always centered on the self in a hedonic way, which can make a person more selfish and thereby have negative effects on the well-being of others (Dambrun and Ricard, 2011). We suffer when egoism craves or clings to things or desires (Dalai Lama, 1995a). Thus, Buddhists tend to argue that the desire-driven pursuit of happiness can lead to negative emotions such as pride and greed, and negative behavior such as cruelty and violence; hence, happiness fluctuates (Dambrun and Ricard, 2011).

According to the NT, the psychological functioning of the self, in contrast to that of the nonself, is concerned mostly with the individual, rather than with the person and the knowledge/wisdom and actions arising from the Buddha's teachings (see Figure 2). This means that the nonself minimizes the individual and maximizes the person, knowledge/wisdom, and good actions as described in Buddhist teachings. Figure 2 illustrates that the bigger size of the individual, knowledge/wisdom, person or action, the greater its impact on the nonself or the self. The MMS circle in the figure refers to the creation of desire by the psychological functions of the individual the nonself state has no desires, thus it has no circle in the figure.

#### Psychological functioning of the nonself and egoism

**Figure 3:**
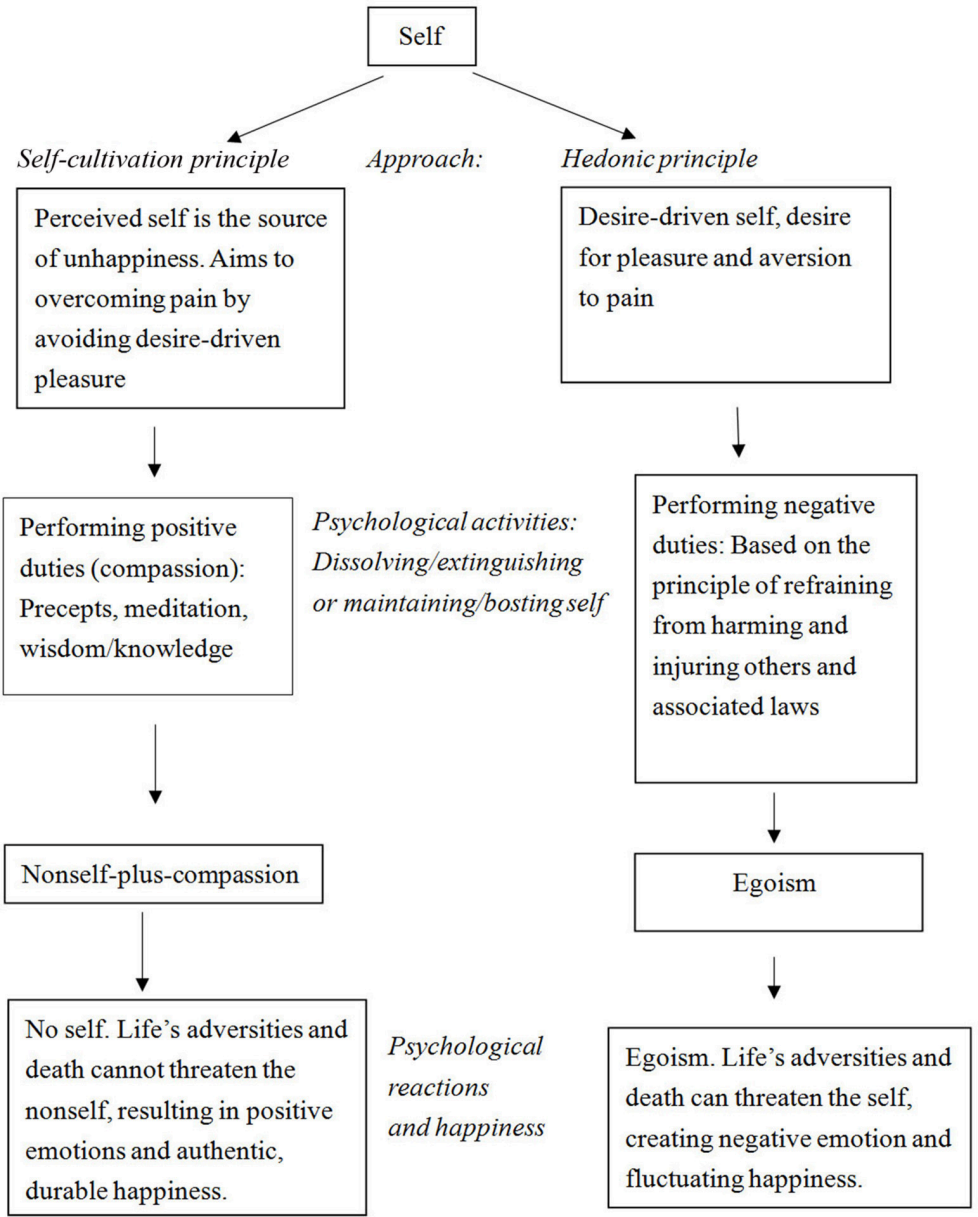
**Psychological functioning of the nonself and the self**.

In this section, I explain how Buddhism helps us to eliminate unhappiness caused by maintaining the self, and how it strengthens the self and elucidates the psychological transition from the self state to the nonself state (see Figure 3). The self engages in psychological activities to strengthen itself by applying the hedonic principle for the purpose of avoiding the pain caused by desire-driven pleasure. However, it does not do so successfully. In contrast, the nonself aims to overcome this suffering directly and does do it successfully. It employs the self-cultivation principle by practicing renunciation of desires, compassion, meditation, and absorbing Buddhist wisdom to see through and overcome the delusion of the self, leading to a profound transformation integrally connected to the experience of eliminating the sense of self and its psychological structures (Dalai Lama, 2005; Albahari, 2014). Finally, one derives authentic and durable happiness as a result of cultivating Buddhist teachings and practices (Wallace and Shapiro, 2006; Albahari, 2014). Egoism employs the hedonic principle in a desire-driven way, leading to negative emotions and fluctuations in happiness (Dambrun and Ricard, 2011). This Buddhist critique of desire-driven pleasure has been indirectly supported by a great deal of recent research demonstrating that the hedonic principle does not predict lasting happiness (Deci and Ryan, 2008; Crespo and Mesurado, 2015).

The psychological processes that represent the self-cultivation approach transitions from the self state to the nonself state by renouncing things we desire (Hwang and Chang, 2009). There is a sense of egolessness that reflects awareness of the (causal) nonself-universe connection as well as compassion and the interdependence and impermanence of all things (Dambrun and Ricard, 2011; Colzato et al., 2012), leading to a sense of no identity. On the contrary, individuals who apply egoism consider themselves as being fundamentally separated from others, autonomous in the world and relatively unique (Dambrun and Ricard, 2011).

Why does perception of the nonself lead to authentic and durable happiness? In addition to the explanations mentioned in earlier section, according to TMT, the prospect of death cannot threaten self-esteem if there is no self or identity, because there is no self-esteem that death can threaten. There would be no anxiety and no unhappiness, but rather the greatest happiness, contentment and equanimity with no capacity to suffer the pain caused by life's adversities. The concept of the nonself provides information about the presence of reliable patterns and coherences in the environment and also helps one cope with these adversities.

## Conclusions

The present research is the first attempt to propose a theory (the NT) based on Buddhist teachings. The universal Mandala Model of Self (MMS) was developed to describe the well-functioning self in various cultures. The end goal of the self is to attain authentic and durable happiness. Because the nonself is considered to be a well-functioning self, the MMS is a suitable basis for constructing the NT. The concepts of egoism and the nonself aspects of psychological self-functioning and their underlying processes are informed by four concepts in MMS: biology, the ideal person, knowledge/wisdom and action. The psychological functioning of the nonself incorporates three ways of the self-cultivation process: giving up desires (by obeying specific precepts), practicing meditation and absorbing Buddhist wisdom. These three ways are essentially ways to experience the reality of emptiness and the importance of compassion, leading to a sense of no identity. The transition from the self state to the nonself state is a deeply transformative experience of eliminating the sense of self and its psychological structures, seeing through and overcoming the illusion of the self. In contrast, the psychological function of egoism is to strengthen the self by applying the hedonic principle to pursue desires leading to fluctuating happiness.

To our knowledge, the NT is the first theory to logically and coherently elucidate the Buddhist teachings in a theoretical way. This is especially true of the three logically interconnected ways in the transition from the self state to the nonself state: obeying precepts, meditating, and absorbing wisdom. Current psychological research offers preliminary confirmation of the Buddhist teachings that can be explained by the NT. For example, many of the emerging Buddhism-related findings demonstrate that mindfulness (Baer, 2003; Brown and Ryan, 2003; Khoury et al., 2015), compassion (Hofmann et al., 2011; Galante et al., 2014; Shonin et al., 2015) and meditation (Cahn and Polich, 2006; Sedlmeier et al., 2012) lead to enhanced positive emotion, attention and subjective well-being. According to the NT, these positive effects can be explained primarily by the concept of the nonself. This is because mindfulness, compassion, and meditation all lead us away from our desires, and thus, from suffering and distractions. This in turn leads to heightened positive emotion, subjective well-being and attentiveness. The NT also offers explanations for the effects of nonself-plus-compassion (Clobert and Saroglou, 2013; Clobert et al., 2015). One of these effects is the promotion of altruism and helping behavior, which according to the NT leads one away from desires and self-centeredness. Moreover, the NT postulates that nonself-plus-compassion conquers the fear of death through a process of self-cultivation that attains the nonself state by minimizing the self state (Shiah and Yit, 2012). The NT provides a novel perspective on the meaning of death. Appealing to nonself-plus-compassion, the NT also provides plausible explanations for mystical and peak experiences. A mystical experience is a sudden, all-encompassing sense of unity underlying all things, often mediated by an experience of light or of pure luminosity, and felt as having its source in a universal love/compassion that eternally pervades the physical universe (Hunt, 2006). A peak experience is that “rare, exciting, oceanic, deeply moving, exhilarating, elevating experience that generates an advanced form of perceiving reality, and are even mystic and magical in their effect upon the experimenter” (Maslow, 1964). In the same way, the NT provides an explanation of the principles of moral conduct overwhelmingly endorsed by most cultures and religions.

### Future directions

Buddhist practice traditionally takes place in the context of spiritual development leading to enlightenment in the form of experience of the nonself, a state of total liberation and authentic and durable happiness (Dalai Lama, 1995a,b). Thus, Buddhist teachings can be used to help people maximize their full human potential. In fact, it has long been believed and testified to that Buddhist teachings and practices successfully remove pain and suffering, implying that they can be used to deal with all psychological problems (Dalai Lama, 1995a,b, 2005). This has practical implications for psychotherapy. Over the past 30 years, a growing number of psychotherapists, counselors and mental health workers have been engaged in various forms of Buddhist psychotherapy. The core idea of Buddhist teachings is not to attach to the self. As noted before, compassion is considered an authentic form of wisdom and a way to attain the state of nonself. The Buddhist construct of (non)attachment, or nonself-plus-compassion, is not inconsistent with concept the Western psychological construal of attachment in the context of certain relationships (Sahdra and Shaver, 2013; Shonin et al., 2014). However, there is a strong and urgent need to ground Buddhist psychotherapy and psychology in more evidence-based research (Kelly, 2008; Shonin et al., 2014). The NT proposed in the present paper provides theoretical direction to the accumulation of such evidence-based data for use in Buddhist psychotherapy. Similarly, Buddhism has adopted an approach to the concept of the self that differs from that in Western psychology. There is a need to construct a nonself psychology based on the emptiness construct.

Obviously, it would take much effort and time to fully describe how one can achieve the ideal state of nonself through Buddhist cultivation and the avoidance of hedonic activities. In the present paper, only a very basic and initial framework of the nonself and the psychological processes that create it have been elucidated. An example is the widely-used and practical mindful-based meditation technique (Khoury et al., 2013, 2015) that forms a foundation of the Buddhist process of self-cultivation. Mindfulness was used as a technique to create enhanced subjective well-being because it was considered to be associated with an optimization of moment-to-moment experience in the West (Brown and Ryan, 2003). However, according to the NT, the main purpose of meditation is to get rid of desire conation and behavior rather than to create the positive effects of mindfulness. More sophisticated and detailed empirical research is needed on procedures for attaining the state of the nonself, including the three ways of obeying precepts, meditating and absorbing Buddhist wisdom.

Though the NT proposed in the present paper is very much in its infancy, it is built on very robust cornerstones of Buddhist teachings that have been practiced and tested for more than 2500 years. It also suggests a number of opportunities for further investigation. First, both contemporary and ancient Buddhist texts describe the “nonself state” in valid and illuminating ways that provide a different perspective on human beings than does the Western concept of personality (Hwang, 2011; Johnson, 2015). For example, answers to the profound questions the NT raises about the true nature of the whole person might lead to a more comprehensive understanding of the meaning of life and its ultimate goals. Secondly, preliminary results (Falb and Pargament, 2013) indicate that application of Buddhist coping mechanisms leads to positive subjective well-being and adjustment. Future research based on the NT should also examine its relationship to the successful implementation of such adjustment and coping strategies. Such research would have important implications for understanding the nature of these adjustment processes and mental health generally. Questions remain about how the nonself develops and what psychological and social conditions support and hinder the fulfillment of the goals specified in the NT. Buddhism's concepts of self, egoism and nonself are complex (Dalai Lama, 1995a; Tsong-Kha-Pa, 2000; Albahari, 2014). In the present paper, to link Buddhism to psychology, I used broad definitions of self, egoism and nonself familiar to psychologists. This approach likewise allowed me to link the NT to both psychological studies and Buddhist teachings. It also demonstrates that the NT is testable using the methods of psychological research. Future studies are needed to elucidate these complex concepts of self, egoism and nonself as well as their implications.

Buddhism offers ideas about how self-cultivation can be used to attain the ultimate state of nonself-plus-compassion. From the moral perspective, nonself-plus-compassion can be regarded as a very high standard of morality and a high level of moral expertise. It has been suggested that this moral expertise emerges from the interactions among beliefs, desires, and moral actions (Hulsey and Hampson, 2014). Little is currently known about these interactions. The NT not only offers insight into how these interactions work, but it also explains why we need moral codes and where they lead us to. For example, according to the NT, moral actions lead us away from desires and suffering and toward the nonself state. This line of future research also could include the effects of obeying the precepts, which is comprised of practicing compassion and acting morally.

Though Buddhism is thought to be practiced in all cultures, it developed and is mainly practiced in Asia. One might ask whether it is uniquely suitable to certain cultures. For example, the Buddhist core concept of avoidance of desire-driven pleasure can be found across cultures (Joshanloo, 2014; Joshanloo and Weijers, 2014). Other concepts similar to nonself-plus-compassion, such as altruism, mindfulness, mediation, mystical/peak experience, death anxiety and moral conduct, are also apparently found across cultures. The NT provides a sophisticated framework to explain a possible mechanism for these universal effects and phenomenon.

## Concluding remarks

There have been very few empirical studies or theories directly targeting Buddhist teachings. This paper is the first to postulate an academically respectable theory based on a full consideration of Buddhist teachings. The hope is that the present research has helped to fill these conceptual gaps, because it suggests that Buddhism provides a reliable and useful way to cope with life's adversities. It guides us toward authentic, durable happiness, and it contributes to the solution of a variety of mental health problems. Thus, the intention of this article was to offer a theory to guide future and innovative research into the potential mutual enrichment of Buddhism and current psychological theory, research, and practice. Although more research is needed on this front, it is hoped that the NT will open significant new avenues for mental health research and unravel the secret of why Buddhism has lasted for thousands of years.

## Author contributions

The author confirms being the sole contributor of this work and approved it for publication.

## Funding

This study was supported by a research grant received from the Ministry of Science and Technology, Taiwan (104-2420-H-017-001-MY3).

### Conflict of interest statement

The author declares that the research was conducted in the absence of any commercial or financial relationships that could be construed as a potential conflict of interest.

## References

[B1] Albahari M. (2006). Analytical Buddhism: The Two-tiered Illusion of Self. Basingstoke: Palgrave.

[B2] Albahari M. (2014). Insight knowledge of no self in Buddhism: An epistemic analysis. Philos. Imprint 14, 1–30.

[B3] Allen P. M. Edwards J. A. McCullough W. (2015). Does karma exist? Buddhism, social cognition, and the evidence for karma. Int. J. Psychol. Relig. 25, 1–17. 10.1080/10508619.2013.879427

[B4] Alt W. (1980). There is no paradox of desire in Buddhism. Philos. East West 30, 521–528. 10.2307/1398976

[B5] Ariyabuddhiphongs V. Jaiwong D. (2010). Observance of the Buddhist five precepts, subjective wealth, and happiness among buddhists in Bangkok, Thailand. Arch. Psychol. Relig. 32, 327–344. 10.1163/157361210x533274

[B6] Baer R. A. (2003). Mindfulness training as a clinical intervention: a conceptual and empirical review. Clin. Psychol. 10, 125–143. 10.1093/clipsy/bpg015

[B7] Brown K. W. Ryan R. M. (2003). The benefits of being present: mindfulness and its role in psychological well-being. J. Pers. Soc. Psychol. 84, 822–848. 10.1037/0022-3514.84.4.822 12703651

[B8] Buddhaghosa (1975). Path of Purification. Kandy: Buddhist Publication Society.

[B9] Burke B. L. Martens A. Faucher E. H. (2010). Two decades of Terror Management Theory: a meta-analysis of mortality salience research. Pers. Soc. Psychol. Rev. 14, 155–195. 10.1177/1088868309352321 20097885

[B10] Cahn B. R. Polich J. (2006). Meditation states and traits: EEG, ERP, and neuroimaging studies. Psychol. Bull. 132, 180–211. 10.1037/0033-2909.132.2.180 16536641

[B11] Clobert M. Saroglou V. (2013). Intercultural non-conscious influences: prosocial effects of Buddhist priming on Westerners of Christian tradition. Int. J. Intercult. Relat. 37, 459–466. 10.1016/j.ijintrel.2012.10.001

[B12] Clobert M. Saroglou V. Hwang K. K. (2015). Buddhist concepts as implicitly reducing prejudice and increasing prosociality. Pers. Soc. Psychol. Bull. 41, 513–525. 10.1177/0146167215571094 25676193

[B13] Cohen A. B. Hill P. C. (2007). Religion as culture: religious individualism and collectivism among American Catholics, Jews, and Protestants. J. Pers. 75, 709–742. 10.1111/j.1467-6494.2007.00454.x 17576356

[B14] Colzato L. S. Zech H. Hommel B. Verdonschot R. van den Wildenberg W. P. M. Hsieh S. (2012). Loving-kindness brings loving-kindness: the impact of Buddhism on cognitive self-other integration. Psychon. Bull. Rev. 19, 541–545. 10.3758/s13423-012-0241-y 22427265

[B15] Crespo R. F. Mesurado B. (2015). Happiness economics, eudaimonia and positive psychology: from happiness economics to flourishing economics. J. Happiness Stud. 16, 931–946. 10.1007/s10902-014-9541-4

[B16] Dalai Lama (1995a). The Path to Enlightenment. Ithaca, NY: Snow Lion.

[B17] Dalai Lama (1995b). The Power of Compassion. London: Thorsons.

[B18] Dalai Lama (2001). Stages of Meditation: Training the Mind for Wisdom. London: Rider.

[B19] Dalai Lama (2005). The Many Ways to Nirvana. London: Mobius.

[B20] Dambrun M. Ricard M. (2011). Self-centeredness and selflessness: a theory of self-based psychological functioning and its consequences for happiness. Rev. Gen. Psychol. 15, 138–157. 10.1037/a0023059

[B21] Deci E. L. Ryan R. M. (2008). Hedonia, eudaimonia, and well-being: an introduction. J. Happiness Stud. 9, 1–11. 10.1007/s10902-006-9018-1

[B22] Eckensberger L. H. (1990). From cross-cultural psychology to cultural psychology. Q. Newsl. Lab. Compar. Hum. Cogn. 12, 37–52.

[B23] Eckensberger L. H. (1996). Agency, action and culture: three basic concepts for cross-cultural psychology, in Asian Contributions to Cross-cultural Psychology, eds Pandey J. Sinha D. Bhawuk D. P. S. (New Delhi: Sage Publications), 72–105.

[B24] Eckensberger L. H. (2012). Culture-inclusive action theory: action theory in dialectics and dialectics in action theory, in Oxford Handbook of Culture and Psychology, ed Valsine J. (Oxford: Oxford University Press), 357–402.

[B25] Falb M. D. Pargament K. I. (2013). Buddhist coping predicts psychological outcomes among end-of-life caregivers. Psycholog. Relig. Spiritual. 5, 252–262. 10.1037/a0032653

[B26] Fink C. K. (2012). The 'scent' of a self: Buddhism and the first-person perspective. Asian Philos. 22, 289–306. 10.1080/09552367.2012.709736

[B27] Fredrickson B. L. Cohn M. A. Coffey K. A. Pek J. Finkel S. M. (2008). Open hearts build lives: positive emotions, induced through loving-kindness meditation, build consequential personal resources. J. Pers. Soc. Psychol. 95, 1045–1062. 10.1037/a0013262 18954193 PMC3156028

[B28] Galante J. Galante I. Bekkers M. J. Gallacher J. (2014). Effect of kindness-based Meditation on health and well-being: a systematic review and meta-analysis. J. Consult. Clin. Psychol. 82, 1101–1114. 10.1037/a0037249 24979314

[B29] Giddens A. (1984). The Constitution of Society: Outline of the Theory of Structuration. Berkeley, CA: University of California Press.

[B30] Giddens A. (1993). New Rules of Sociological Method: A Positive Critique of Interpretative Sociologies, 2nd Edn. Stanford: Stanford University Press.

[B31] Gilbert P. (2009). Introducing compassion-focused therapy. Adv. Psychiatr. Treat. 15, 199–208. 10.1192/apt.bp.107.005264

[B32] Giles J. (1993). The no-self theory, hume, buddism, and personal identity. Philos. East West 43, 175–200. 10.2307/1399612

[B33] Greenberg J. Pyszczynski T. Solomon S. Rosenblatt A. Veeder M. Kirkland S. (1990). Evidence for terror management theory II: the effects of mortality salience on reactions to those who threaten or bolster the cultural worldview. J. Pers. Soc. Psychol. 58, 308–318. 10.1037/0022-3514.58.2.308

[B34] Greenberg J. Simon L. Pyszczynski T. Solomon S. Chatel D. (1992). Terror management and tolerance: does mortality salience always intensify negative reactions to others who threaten one's worldview? J. Pers. Soc. Psychol. 63, 212–220. 10.1037/0022-3514.63.2.212 1403612

[B35] Greenberg J. Soloman S. Pyszczynski T. (1986). The cause and consequences of a need for self-esteem. A terror management theory, in Public Self and Private Self, ed Baunmeister R. F. (New York, NY: Springer-Verlag), 189–192.

[B36] Hallam W. T. Olsson C. A. O'Connor M. Hawkins M. Toumbourou J. W. Bowes G. (2014). Association between adolescent eudaimonic behaviours and emotional competence in young adulthood. J. Happiness Stud. 15, 1165–1177. 10.1007/s10902-013-9469-0

[B37] Harris G. G. (1989). Concepts of individual, self, and person in. Am. Anthropol. 91, 599–612. 10.1525/aa.1989.91.3.02a00040

[B38] Heintzelman S. J. King L. A. (2014). (The feeling of) meaning-as-information. Pers. Soc. Psychol. Rev. 18, 153–167. 10.1177/1088868313518487 24501092

[B39] Hofmann S. G. Grossman P. Hinton D. E. (2011). Loving-kindness and compassion meditation: psychological interventions. Clin. Psychol. Rev. 31, 1126–1132. 10.1016/j.cpr.2011.07.003 21840289 PMC3176989

[B40] Hulsey T. L. Hampson P. J. (2014). Moral expertise. New Ideas Psychol. 34, 1–11. 10.1016/j.newideapsych.2014.02.001

[B41] Hunt H. (2006). The truth value of mystical experience. J. Conscious. Stud. 13, 5–43.

[B42] Huta V. Waterman A. S. (2014). Eudaimonia and its distinction from hedonia: developing a classification and terminology for understanding conceptual and operational definitions. J. Happiness Stud. 15, 1425–1456. 10.1007/s10902-013-9485-0

[B43] Hwang K.-K. (2011). The Mandala model of self. Psychol. Stud. 56, 329–334. 10.1007/s12646-011-0110-1

[B44] Hwang K.-K. Chang J. (2009). Self-cultivation culturally sensitive psychotherapies in Confucian societies. Counsel. Psychol. 37, 1010–1032. 10.1177/0011000009339976

[B45] Jaffe A. (1964). Symbolism in the visual arts, in Man and His Symbols, ed Jung C. G. (New York, NY: Dell Pub. Co), 230–271.

[B46] Johnson E. L. (2015). Mapping the field of the whole human: toward a form psychology. New Ideas Psychol. 38, 4–24. 10.1016/j.newideapsych.2013.05.005

[B47] Joshanloo M. (2014). Eastern conceptualizations of happiness: fundamental differences with Western views. J. Happiness Stud. 15, 475–493. 10.1007/s10902-013-9431-1

[B48] Joshanloo M. Weijers D. (2014). Aversion to happiness across cultures: a review of where and why people are averse to happiness. J. Happiness Stud. 15, 717–735. 10.1007/s10902-013-9489-9

[B49] Kelly B. D. (2008). Buddhist psychology, psychotherapy and the brain: a critical introduction. Transcult. Psychiatr. 45, 5–30. 10.1177/1363461507087996 18344250

[B50] Khoury B. Lecomte T. Fortin G. Masse M. Therien P. Bouchard V.. (2013). Mindfulness-based therapy: a comprehensive meta-analysis. Clin. Psychol. Rev. 33, 763–771. 10.1016/j.cpr.2013.05.005 23796855

[B51] Khoury B. Sharma M. Rush S. E. Fournier C. (2015). Mindfulness-based stress reduction: a meta-analysis. J. Psychosom. Res. 78, 519–528. 10.1016/j.jpsychores.2015.03.009 25818837

[B52] Klein S. B. (2014). Sameness and the self: philosophical and psychological considerations. Front. Psychol. 5:29. 10.3389/fpsyg.2014.00029 24523707 PMC3905202

[B53] Lee B.-L. Hwang K.-K. Shiah Y.-J. (2015a). To construct a grief healing theory in Confucian societies: a Confucian harmonious relationships approach of grief healing theory. J. Counsel. Psychol. Rehabil. Counsel. 28, 7–33. (in Chinese). 10.6308/JCPRC.28.01

[B54] Lee Y.-H. Shiah Y.-J. Chen S. C.-J. Wang S.-F. Young M.-S. Hsu C.-H.. (2015b). Improved emotional stability in experienced meditators with concentrative meditation based on electroencephalography and heart rate variability. J. Altern. Complement. Med. 21, 31–39. 10.1089/acm.2013.0465 25354314

[B55] Leifer R. (1999). Buddhist conceptualization and treatment of anger. J. Clin. Psychol. 55, 339–351. 10.1002/(sici)1097-4679(199903)55:3<339::aid-jclp6>3.0.co;2-e 10321748

[B56] Lippelt D. P. Hommel B. Colzato L. S. (2014). Focused attention, open monitoring and loving kindness meditation: effects on attention, conflict monitoring, and creativity: a review. Front. Psychol. 5:5. 10.3389/fpsyg.2014.01083 25295025 PMC4171985

[B57] Lutz A. Greischar L. L. Rawlings N. B. Ricard M. Davidson R. J. (2004). Long-term meditators self-induce high-amplitude gamma synchrony during mental practice. Proc. Natl. Acad. Sci. U.S.A. 101, 16369–16373. 10.1073/pnas.0407401101 15534199 PMC526201

[B58] Lutz A. Slagter H. A. Dunne J. D. Davidson R. J. (2008). Cognitive-emotional interactions: attention regulation and monitoring in meditation. Trends Cogn. Sci. 12, 163–169. 10.1016/j.tics.2008.01.005 18329323 PMC2693206

[B59] MacKenzie M. (2010). Enacting the self: Buddhist and enactivist approaches to the emergence of the self. Phenomenol. Cogn. Sci. 9, 75–99. 10.1007/s11097-009-9132-8

[B60] Maslow A. H. (1964). Religions, Values, and Peak Experiences. London: Penguin Books Limited.

[B61] Michalon M. (2001). “Selflessness” in the service of the ego: Contributions, limitations and dangers of Buddhist psychology for western psychotherapy. Am. J. Psychother. 55, 202–218. 11467257 10.1176/appi.psychotherapy.2001.55.2.202

[B62] Murguia E. Diaz K. (2015). The philosophical foundations of cognitive behavioral therapy: Stoicism, Buddhism, Taoism, and Existentialism. J. Evid. Based Psychother. 15, 37–50.

[B63] Nickerson A. Hinton D. E. (2011). Anger regulation in traumatized Cambodian refugees: the perspectives of Buddhist monks. Cult. Med. Psychiatry 35, 396–416. 10.1007/s11013-011-9218-y 21630119

[B64] Number of Buddhists worldwide (2015). Retrieved from: http://www.buddhanet.net/e-learning/history/bud_statwrld.htm (Retrieved December 24, 2015).

[B65] Nyanaponika N. T. (1998). Abhidhamma Studies: Buddhist Explorations of Consciousness and Time, 4th Edn. Boston, MA: Wisdom Press.

[B66] Oyserman D. Coon H. M. Kemmelmeier M. (2002). Rethinking individualism and collectivism: evaluation of theoretical assumptions and meta-analyses. Psychol. Bull. 128, 3–72. 10.1037//0033-2909.128.1.3 11843547

[B67] Park C. L. (2010). Making sense of the meaning literature: an integrative review of meaning making and its effects on adjustment to stressful life events. Psychol. Bull. 136, 257–301. 10.1037/a0018301 20192563

[B68] Pyszczynski T. Solomon S. Greenberg J. Arndt J. Schimel J. (2004). Why do people need self-esteem? A theoretical and empirical review. Psychol. Bull. 130, 435–468. 10.1037/0033-2909.130.3.435 15122930

[B69] Rinpoche S. (1998). The Tibetan Book of Living and Dying. London: Rider.

[B70] Sahdra B. K. Shaver P. R. (2013). Comparing attachment theory and Buddhist psychology. Int. J. Psychol. Relig. 23, 282–293. 10.1080/10508619.2013.795821

[B71] Salgado N. S. (2004). Religious identities of Buddhist nuns: training precepts, renunciant attire, and nomenclature in theravada Buddhism. J. Am. Acad. Relig. 72, 935–953. 10.1093/jaarel/lfh084

[B72] Sedlmeier P. Eberth J. Schwarz M. Zimmermann D. Haarig F. Jaeger S.. (2012). The psychological effects of meditation: a meta-analysis. Psychol. Bull. 138, 1139–1171. 10.1037/a0028168 22582738

[B73] Sheng Yen S. (1999). Essentials of Buddhist Sila and Vinaya. Taipei: Dharma Drum Corp. (in Chinese).

[B74] Shiah Y.-J. Yit K.-T. (2012). Adopting the Buddhist concept of nature of death and life impermanence in investigating the death terror. Indigenous Psychol. Res. Chin. 36, 167–189. (in Chinese). 10.6254/2012.38.167

[B75] Shonin E. Van Gordon W. Compare A. Zangeneh M. Griffiths M. D. (2015). Buddhist-derived loving-kindness and compassion meditation for the treatment of psychopathology: a systematic review. Mindfulness 6, 1161–1180. 10.1007/s12671-014-0368-1

[B76] Shonin E. Van Gordon W. Griffiths M. D. (2014). The emerging role of Buddhism in clinical psychology: toward effective integration. Psycholog. Relig. Spiritual. 6, 123–137. 10.1037/a0035859

[B77] Steger M. F. Kashdan T. B. Oishi S. (2008). Being good by doing good: daily eudaimonic activity and well-being. J. Res. Pers. 42, 22–42. 10.1016/j.jrp.2007.03.004

[B78] Sundararajan L. (2008). Toward a reflexive positive psychology: insights from the Chinese Buddhist notion of emptiness. Theory Psychol. 18, 655–674. 10.1177/0959354308093400

[B79] Triandis H. C. (2001). Individualism-collectivism and personality. J. Pers. 69, 907–924. 10.1111/1467-6494.696169 11767823

[B80] Triandis H. C. Gelfand M. J. (1998). Converging measurement of horizontal and vertical individualism and collectivism. J. Pers. Soc. Psychol. 74, 118–128. 10.1037/0022-3514.74.1.118

[B81] Tsong-Kha-Pa (2000). The Great Treatise on the Stages of the Path to Enlightenment (Trans. by: T. Lamrin Chenmo Translation Committee). Ithaca, NY: Snow Lion Publications.

[B82] Wada K. Park J. (2009). Integrating Buddhist psychology into grief counseling. Death Stud. 33, 657–683. 10.1080/07481180903012006 19623766

[B83] Wallace B. A. (2001). Intersubjectivity in Indo-Tibetan Buddhism. J. Conscious. Stud. 8, 209–230.

[B84] Wallace B. A. Shapiro S. L. (2006). Mental balance and well-being: building bridges between Buddhism and western psychology. Am. Psychol. 61, 690–701. 10.1037/0003-066x.61.7.690 17032069

